# Identification of technical item flaws leads to improvement of the quality of single best Multiple Choice Questions

**DOI:** 10.12669/pjms.293.2993

**Published:** 2013

**Authors:** Humaira Fayyaz Khan, Khalid Farooq Danish, Azra Saeed Awan, Masood Anwar

**Affiliations:** 1 Dr. Humaira Fayyaz Khan, MBBS, FCPS, Assistant Professor Physiology, Islamic International Medical College, Rawalpindi, Pakistan.; 2 Dr. Khalid Farooq Danish, MBBS, FCPS, Professor and Head of Surgery, Islamic International Medical College, Rawalpindi, Pakistan.; 3 Dr. Azra Saeed Awan, MBBS, FCPS, Professor of Gynaecology and Obstetrics, Islamic International Medical College, Rawalpindi, Pakistan.; 4 Maj. Gen.(R) Masood Anwar, MBBS, FCPS, Principal, Islamic International Medical College, Rawalpindi, Pakistan.

**Keywords:** Frequency, Item writing flaws, Testwiseness

## Abstract

***Objective:*** The purpose of the study was to identify technical item flaws in the multiple choice questions submitted for the final exams for the years 2009, 2010 and 2011.

***Methods:*** This descriptive analytical study was carried out in Islamic International Medical College (IIMC). The Data was collected from the MCQ’s submitted by the faculty for the final exams for the year 2009, 2010 and 2011. The data was compiled and evaluated by a three member assessment committee. The data was analyzed for frequency and percentages the categorical data was analyzed by chi-square test.

***Results:*** Overall percentage of flawed item was 67% for the year 2009 of which 21% were for testwiseness and 40% were for irrelevant difficulty. In year 2010 the total item flaws were 36% and 11% testwiseness and 22% were for irrelevant difficulty. The year 2011 data showed decreased overall flaws of 21%. The flaws of testwisness were 7%, irrelevant difficulty were 11%.

***Conclusion:*** Technical item flaws are frequently encountered during MCQ construction, and the identification of flaws leads to improved quality of the single best MCQ’s.

## INTRODUCTION

Altering the mode of examination to single best question type represents a major challenge for a faculty in any medical college. With change in the curriculum, the modality of assessment also changes. The new system of examination focuses on application, problem solving and integration of the different concepts taught. In Islamic International Medical College the mode of examination based on one best question is being practiced since 2009.

MCQs or single best questions are difficult and time-consuming to construct, even for those who have been formally trained in the construction of MCQs.^1^ Properly made MCQs leads to impartial testing of the student that can measure knowledge, comprehension, application and analysis.^2^^,^^3^

Characteristics of effective MCQs can be described in terms of the overall item, the stem, and the options. The stem generally consist of a clinical case presentation and a lead-in question, followed by a series of choices, typically one correct/best answer and four distractors.^4^ Questions that aim to assess really important topics cannot do so unless they are well-structured i.e. avoiding flaws that benefit the testwise examinee; those students who answer questions alone on their test taking skills and not on their amount of expertise on the subject that is being covered.^5^ Also avoiding irrelevant difficulty are prerequisites that must be met in order for test questions to generate valid scores.^5^

Outlines regarding effective item-writing have been documented; however manipulations of these principles are very common in medical education with resultant flawed item questions.^3^^,^^4^ Flawed MCQs interfere with accurate and meaningful interpretation of test scores and negatively impact student pass rates. One aspect where many MCQs fail is in having effective distractors.^5^ Teachers often spend a great deal of time constructing the stem and much less time on developing plausible options to the correct answer.^5^

Two types of technical item flaws: testwiseness and irrelevant difficulty are described in literature. Flaws related to testwiseness make it easier for some students to answer the question correctly, based on their test-taking skills alone.^4^^,^^5^ Flaws related to irrelevant difficulty make the question difficult for reasons unrelated to the trait that is the focus of assessment. The increased test and item difficulty associated with the use of flawed items lead to artificial difficulty to the test scores.^6^

The purpose of this study was to examine structural concerns which are important for the formation of high-quality test questions. Thus the main objective of this study was to identify common technical flaws in assessment items encountered during the paper setting of examinations of 2009 to 2011.

## METHODS

This descriptive study was conducted at Riphah University Rawalpindi after the completion of assessment for the year 2011. There were no human subjects involved in the study. Therefore it was exempted for obtaining an ethical approval certificate.

The assessment data for the years 2009, 2010 and 2011 was collected and reviewed. These items were reviewed by a three member assessment committee. The original single best choice questions that had been submitted to the assessment committee for the purpose of exams were grouped according to the year and were then analyzed for technical item flaws.

***Inclusion criteria: ***All questions submitted for the years 2009, 2010 & 2011. During analysis intrinsic structure of the question was checked for technical accuracy. Items were classified as 'flawed' if they contained one of the flaws. Frequently observed flaws were grouped into:

1- Issues Related to Testwiseness

- Grammatical Cues and errors
- Logical cues
- Use of absolute (e.g. using *often, sometimes* in MCQ) terms.^5^
- Long correct answer
- Convergence strategy

2- Issues related to irrelevant difficulty

- All except or none except in the stem.
- Question’s containing negative statement of MCQ’s.
- All of the above or none of the above in the options.
- Heterogeneous options.
- Numeric data not stated consistently.

3- Moreover, the papers were corrected for spelling, punctuation, grammar and terminology by the assessment committee. Total of items reviewed were calculated. Percentages of the technical flaws encountered were calculated with measurement of frequencies. Chi-square analysis was used to analyze the improvement in categories of variables between the years. The data of each was analyzed using SPSS 13.

## RESULTS

Overall 4550 MCQ’s of single best type and a total of 20,000 options were analyzed for item flaws using guidelines given in “constructing written test questions for the basic and clinical science” by National Board of Medical Examiners were evaluated by the assessment committee. The flaws of these MCQ items were broadly classified into four types of flaws Table-I. 

Analysis of the results showed that a total number of 850 MCQs were assessed for the year 2009. This year questions examined were for one class. The overall percentage of the flawed items in this year was 67%. Further analysis showed that the proportion of flaws related to testwiseness was 21%, 40% of the items had flaws of irrelevant difficulty, 2.5% punctuation errors and 3.3% spelling mistakes.

For the year 2010 there were 1500 MCQ’s which were assessed for two years. The total flaws observed in this year were 36%. The flaws related to testwisness were 11%, irrelevant difficulty were 22%, punctuation error and spelling mistakes were 1.3% and 1% percent respectively.

Analysis showed that in the year 2011 data for three classes was analyzed. The study of questions showed that overall flaws encountered were 21%. The flaws of testwisness were 7%, irrelevant difficulty were 11%, punctuation errors were 1% and spelling mistakes were 1.2%. 

## DISCUSSION

Paper setting and assessment designing, consisting of MCQs is a complex process. It is important to recognize its potential strength which is a broad coverage of concepts that can be tested consistently. A well- set paper for assessment reflects positively on a curriculum which has been taught. It proves to the students that the curriculum’s supervisor and the teaching staff take pride in all aspects of the course.

While much has been written in context of developing a good MCQ, there is very little actual data concerning the analysis of a MCQ. The results in this study show that the frequency of item flaws encountered in the year 2009 were 67% which is comparable to flaws encountered in a study conducted by Ellsworth et al in psychology test banks.^7^ Another, study by Hansen in an accounting test banks found item flaws to be 75%.^8^ In the year 2010, the total item flaws were 36%. This is comparable to a study by Downing’s who had conducted a study in medical college exams and found that 46% of MCQs contained item-writing violations. The frequency of item writing flaws found in MCQs in a study by Tarrant et al (2005) is 46.2% which is a study conducted on nursing curriculum.^9^ However, the evaluation of the data from 2011 showed that overall the total item flaws were 21%. This is substantially less as compared to data seen from the year 2009 and 2010.

In the present, study a number of violations were found that help students correctly answer questions based on cues given in the stem or the options, rather than knowledge. Item writing flaws (IWFs) such as longest correct option, logical cues, word repeats, use of ‘‘all of the above,’’ and use of absolute terms make MCQs easier by providing helpful cues to students as to what is the correct answer. MCQ’s with heterogenous options apparently increase the difficulty of a question and deal with miscellaneous facts.^4^^,^^5^

This study analyzed these item flaws encountered during the analysis of item submitted across three years. The frequency of flaws related to testwiseness were 21% and those which were correlated to irrelevant difficulty were 40% for the year 2009. This percentage of item flaws is comparable to a study by Danish in 2010 which had similar percentage of item flaws.^10^ Though that study was analysis of data from module exams that were conducted during the academic year, the proportion of the flaws related to testwiseness steadily decreased in 2010 and 2011.

The identification of these item writing flaws also highlights the fact that the faculty members preparing these items should be trained in a faculty development workshop.^11^ Research in other disciplines has shown that training improves the quality of MCQs developed by teaching faculty.^12^ Despite the fact that in the year 2009, the faculty had been trained for the MCQ writing, lack of practice was a recognizable factor contributing to the high number of errors in items analyzed for the year 2009 and 2010. As the faculty preceded from 2009 into 2010 and then 2011, the level of MCQ construction and item writing skills of the faculty improved. Moreover, relevant feedback regarding removal of errors give good results in improving the quality of items prepared by the faculty members.

**Table I:** Categories of Item flaws encountered

*Sr. No*	*Category*	*Sub- category/flaws*
1.	Issues related to testwiseness	Grammatical Cues and errors. Logical cues Use of absolute (e.g. using *often, sometimes* in MCQ) terms. Long correct answer. Convergence strategy
2.	Issues related to irrelevant difficulty	All except or none except in the stem. Question’s containing negative statement of MCQ’s All of the above or none of the above in the options. Heterogeneous options. Numeric data not stated consistently.
3.	Punctuation errors	Grammer, capitalization &use of punctuation symbols.
4.	Spelling mistake	Correct spellings given in the text books of medicine

**Table-II:** Analysis of technical item flaws

*Technical Item Flaws*	*Year 2009*	*Year 2010*	*Year 2011*
No of MCQ’s	850	1500	2150
Issues Related to Testwiseness	180(21%)	165(11%)	155(7%)
Issues related to irrelevant difficulty	340(40%)	330(22%)	243(11%)
Punctuation errors	23(2.5%)	20(1.3%)	22(1.1%)
Spelling mistakes	30(3.3%)	30(2%)	30(1.35%)
Total item flaws	673 (67%)	545(36.3%)	450(21%)

It is also suggested to the faculty members that in order to combat the issues of irrelevant difficulty, while preparing for lecture the relevant MCQ be prepared at the same time. Most often important assessment are written and assembled at the last moment. While the faculty members take lecturing seriously few make an effort to prepare the assessment. Furthermore, by the time items are collected from half a dozen or more lecturers who may have been involved in teaching there is inadequate time or opportunity to review before being submitted to the assessment committee.

For planning an effective assessment it is emphasized that the items prepared by the faculty members should be carefully analyzed before they are put in an evaluation paper. The current research points in detail the types of mistake that are mainly committed in the construction of MCQs. It also gives guidelines for authors of the MCQ items about the common error committed during the preparation of MCQ’s.

If an MCQ is going to be used to assess higher order cognitive skills, there needs to be a process in place where adequate instruction and feedback is given to the item authors. The results verify that with repetition and practice the standard of MCQ for assessment paper’s can be improved. To ensure better quality of MCQ it is suggested that the items before being submitted to the assessment committee should be evaluated at inter departmental level and then submitted to the finalizing committee. This will lead to better written items and save time as well.

## CONCLUSION

Technical item flaws are frequently encountered during MCQ construction, and the identification of these flaws leads to improved quality of the single best MCQ’s. In order to rectify these flaws the faculty should be trained in item writing skills. While on the spot training can be done at the time of assessment but better results can be obtained if the faculty is trained prior to the final exams.
